# Chondroitin Sulfate Is Required for Onset and Offset of Critical Period Plasticity in Visual Cortex

**DOI:** 10.1038/s41598-017-04007-x

**Published:** 2017-10-03

**Authors:** Xubin Hou, Nozomu Yoshioka, Hiroaki Tsukano, Akiko Sakai, Shinji Miyata, Yumi Watanabe, Yuchio Yanagawa, Kenji Sakimura, Kosei Takeuchi, Hiroshi Kitagawa, Takao K. Hensch, Katsuei Shibuki, Michihiro Igarashi, Sayaka Sugiyama

**Affiliations:** 1 Lab of Neuronal Development, Graduate School of Medical and Dental Sciences, Niigata University, Niigata, 951-8510 Japan; 2 Department of Neurochemistry and Molecular Cell Biology, Graduate School of Medical and Dental Sciences, Niigata University, Niigata, 951-8510 Japan; 3 Trans-disciplinary Research Program, Niigata University, Niigata, 951-8510 Japan; 4 Department of Neurophysiology, Brain Research Institute, Niigata University, Niigata, 951-8585 Japan; 5 Department of Biochemistry, Kobe Pharmaceutical University, Kobe, 658-8558 Japan; 6 Department of Genetic and Behavioral Neuroscience, Graduate School of Medicine, Gunma University, Gunma, 371-8511 Japan; 7 Department of Cellular Neurobiology, Brain Research Institute, Niigata University, Niigata, 951-8585 Japan; 8 Department of Medical BiologyNagakute Japan, Aichi Medical University, Nagakute, Japan; 9 Department of Neurology, Children’s Hospital Boston, Harvard Medical School, Boston, 02115 MA USA; 10 Present Address: Division of Neurobiology and Anatomy, Graduate School of Medical and Dental Sciences, Niigata University, Niigata, Japan; 11 Present Address: Bioscience and Biotechnology Center, Nagoya University, Nagoya, Japan; 12 Present Address: Department of Preventive Medicine, Graduate School of Medical and Dental Sciences, Niigata University, Niigata, Japan

## Abstract

Ocular dominance plasticity is easily observed during the critical period in early postnatal life. Chondroitin sulfate (CS) is the most abundant component in extracellular structures called perineuronal nets (PNNs), which surround parvalbumin-expressing interneurons (PV-cells). CS accumulates in PNNs at the critical period, but its function in earlier life is unclear. Here, we show that initiation of ocular dominance plasticity was impaired with reduced CS, using mice lacking a key CS-synthesizing enzyme, *CSGalNAcT1*. Two-photon *in vivo* imaging showed a weaker visual response of PV-cells with reduced CS compared to wild-type mice. Plasticity onset was restored by a homeoprotein Otx2, which binds the major CS-proteoglycan aggrecan and promotes its further expression. Continuous CS accumulation together with Otx2 contributed bidirectionally to both onset and offset of plasticity, and was substituted by diazepam, which enhances GABA function. Therefore, CS and Otx2 may act as common inducers of both onset and offset of the critical period by promoting PV-cell function throughout the lifetime.

## Introduction

Neural circuits are refined by experience at an early stage of postnatal life. Ocular dominance plasticity is a well-studied paradigm for experience-dependent cortical development^1^. Monocular deprivation (MD) yields a strong shift in cortical responsiveness toward the non-deprived eye^2^, concomitant with remodeling of dendritic spines and axons during the critical period. Hence, MD produces a permanent deficit in deprived-eye vision known as amblyopia^3^.

The onset of the critical period occurs with maturation of cortical GABA circuits^4,5^. Among multiple subsets of GABAergic interneurons, accumulating evidence indicates the involvement of fast-spiking, parvalbumin-expressing basket cells (PV-cells). These cells provide somatic inhibition to regulate the response gain of their target neurons^6^ and respond according to an imbalance in visual experience, triggering plasticity^7–9^. Distinct molecular inducers of PV-cell maturation such as brain-derived neurotrophic factor (BDNF) and Otx2 homeoprotein prematurely accelerate the critical period^10,11^. Conversely, immature PV circuits in mice lacking Otx2 or neuronal activity-regulated pentraxin (Narp) fail to activate the onset of the critical period^11,12^.

Cortical plasticity associated with amblyopia declines after onset. Previous reports showed the involvement of inhibitory circuits in restriction of plasticity later in life. Re-initiation of plasticity is triggered by reducing inhibition^13–15^. Disrupting neuromodulator activity by neurotransmitters such as serotonin or acetylcholine also re-initiates plasticity due to insufficient inhibitory activity^16,17^. Converging evidence indicates that weakened inhibition resets the excitatory-inhibitory balance, similar to before the critical period, and then plasticity is reinforced during recovery.

Adult plasticity following prolonged MD facilitates potentiation of open eye responses without amblyopic depression of responses to the closed eye^18^. Inhibitory circuits such as vasoactive intestinal peptide (VIP)- or somatostatin (SST)-positive interneurons are required for adult plasticity, whereas the requirement for PV-cells is controversial^19,20^. Thus, the critical period and adult plasticity may be mediated by distinct mechanisms. However, whether maturity of PV circuits is specifically required for restriction of the critical period as well as its activation is still unclear.

Components of perineuronal nets (PNNs) are considered physical barriers against cortical plasticity^21,22^. PNNs contain abundant chondroitin sulfate (CS) chains, which bind to Otx2 via a glycosaminoglycan (GAG)-binding motif^23,24^. PNNs capture this homeoprotein specifically in PV-cells, and this mechanism of Otx2 uptake promotes further formation of PNNs^11,23^. Acute removal of Otx2 reactivates the plasticity for amblyopia in the adult cortex, suggesting a role for Otx2 as molecular brakes against critical period plasticity^23,25^. In contrast to the effect of Otx2 on both onset and offset of plasticity, impairment of PNNs inhibits plasticity offset^21,22,24^, but whether impairment of PNNs also affects its onset is not known. The concept of a molecular brake was derived from inhibiting the plasticity^26^, suggesting a different molecular mechanism between initiation and termination of the critical period. However, the possibility that the same molecules act to modulate both onset and offset of plasticity cannot be ruled out.

CS is a GAG that consists of repeating disaccharide units of *N*-acetylgalactosamine (GalNAc) and glucuronic acid residues^27^. The synthesizing enzyme CS *N*-acetylgalactosaminyltransferase-1 (T1) catalyzes the rate-limiting step in CS synthesis, which involves transfer of GalNAc to the linker of the core protein of proteoglycans^28,29^. Because CS proteoglycans (CSPGs) are expressed before the onset of the critical period with visual experience^21,22,30^, optimizing CS synthesis may be implicated in both initiation and termination of plasticity.

To address the role of CS synthesis in plasticity, we used T1 knockout (KO) mice, which have a slightly smaller body size but no gross brain abnormalities^31^. Here, we show that reduction in CS biosynthesis inhibited PV-cell maturation and consequently prevented the onset of plasticity. Enhancing GABAergic functions with diazepam (DZ) restored the plasticity, which did not decline unless additional DZ was administered. Thus, CS plays a bidirectional role in cortical plasticity, likely by post-transcriptional control of Otx2. These results demonstrate that both the onset and offset of plasticity require PV-cell function enhanced by CS accumulation, indicating the importance of optimizing the amount of CS during plasticity.

## Results

### Reduction in CS biosynthesis with T1 deficiency

**Figure 1:**
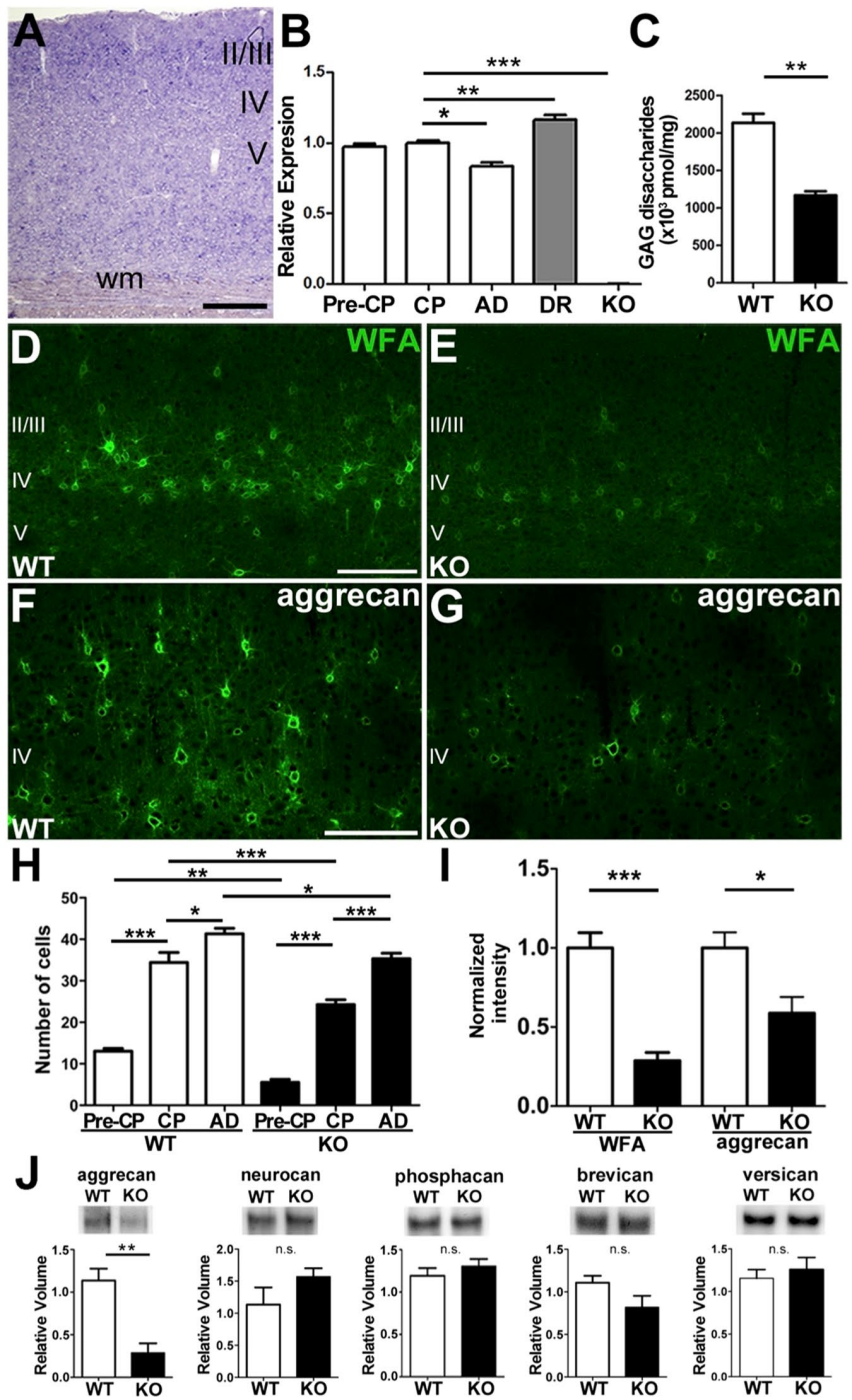
T1 deletion inhibits CS biosynthesis. (**A**,**B**) Expression of *T1* mRNA in mouse V1 (**A**) during postnatal development (**B**, mean and SEM; critical period (CP, P28–P30) versus adulthood (AD, >P60), *p* = 0.02, ANOVA). Dark-rearing (DR) enhances the expression (*p* = 0.007, ANOVA), and genetic *T1* deletion (KO) removes it completely (*p* = 0.0002, ANOVA). II/III, VI, and V indicate the cortical layers; Wm, white matter. The scale bar represents 200 μm. (**C**) Reduction of the total CS amount in the adult V1 of T1 KO mice (mean ± SEM [pmol/mg]; WT 2134.4 ± 123.2 versus KO 1169.5 ± 52.9, four mice, *p* = 0.002, *t*-test). (**D**–**I**) Decreased expression of aggrecan in V1 of T1 KO mice. WFA labeling or aggrecan immunoreaction in the binocular zone was decreased in adult T1 KO (**E**,**G**) compared to WT mice (**D**,**F**). Quantitative analysis of the number of WFA-labeled cells (**H**, 600 × 350 μm area; WT 34.4 ± 2.4, KO 24.3 ± 1.2 for P28–30; WT 41.3 ± 1.3, KO 35.3 ± 1.3 for >P60; 3–4 mice, P28–P30 versus >P60, *p* < 0.0001 for WT, *p* < 0.05 for KO, ANOVA), and WFA-labeled or aggrecan immunofluorescence intensity in the adult V1 (**I**, WFA; three mice, *p* = 0.0008, *t*-test, aggrecan; three mice, *p* = 0.016, *t*-test). (**J**) Expression of CSPGs in the adult V1. Western blotting analyses verified reduction of aggrecan in T1 KO mice (six mice, *p* = 0.005, *t*-test) and no significant difference in the protein quantity of other CSPGs between genotypes (six mice, *p* > 0.05, *t*-test). The full-length blots are shown in a Supplementary Information.

In wild-type (WT) mice, T1 was broadly expressed in the primary visual cortex (V1) as observed using *in situ* hybridization (Fig. 1A). Consistent with early postnatal expression of other PNN components^22^, T1 transcripts were detected using qRT-PCR prior to onset of the critical period (P16–18), were maintained until the peak of the critical period (P28–30), and then were significantly reduced in adulthood (>P60; Fig. 1B). Notably, the expression was elevated after dark rearing from birth to P28, suggesting a reduction in T1 transcripts by experience-dependent maturation of V1. In contrast, T1 was not detected in T1 KO mice.

To confirm whether T1 is responsible for CS synthesis in V1, we biochemically characterized CS that is post-translationally attached to a core protein of proteoglycans^27^. Similar to the reduction in cartilage CS in T1 KO mice^31^, the total amount of CS in V1 was reduced by approximately half compared to the WT (Fig. 1C: four mice, *p* = 0.002, *t*-test). Analysis of the disaccharide composition showed slight changes in the CS sulfation pattern (Table 1), suggesting that the reduction in CS synthesis affected the sulfation pattern in PNNs. During PNN formation in V1, impaired CS biosynthesis resulted in a weakened fluorescence intensity of *Wisteria floribunda* agglutinin (WFA)-labeled GAGs by more than half (Fig. 1D, E, I) and a reduced number of labeled cells in supragranular layers of the binocular zone (Fig. 1H: WT versus KO, 3–4 mice, *p* < 0.01 for P16–18; *p* < 0.0001 for P28–30; *p* < 0.05 for >P60, ANOVA). Notably, the reduction in the total amount of CS and fluorescence intensity of WFA signals were more pronounced than the decreased number of WFA-labeled cells. In both biochemical and histological analyses, CS chains attached to core proteins as the components of proteoglycans were reduced almost by half with T1 deficiency in V1. Thus, we conclude that T1 is a key enzyme in CS synthesis and regulates CS accumulation in PNNs from an early age.

**Table 1:** Disaccharide composition of visual cortical CS.

Disaccharides	WT (n = 4)	KO (n = 4)
ΔDi-0S	9.0 ± 0.18	10.7 ± 0.16 (↑)***
ΔDi-6S	2.7 ± 0.01	2.3 ± 0.03 (↓)***
ΔDi-4S	86.4 ± 0.18	84.6 ± 0.19 (↓)***
ΔDi-diS_D_	0.7 ± 0.03	0.4 ± 0.03 (↓)***
ΔDi-diS_E_	1.24 ± 0.01	1.9 ± 0.03 (↑)***
Total (pmol/mg)%	100.0	100.0

The values (mean and SEM) are expressed as pmol of disaccharide per mg of dried cortical homogenate. Abbreviations: ΔDi-0S, DHexUAa1–3GalNAc; ΔDi-6S, ΔHexUAa1–3GalNAc (6-O-sulfate); ΔDi-4S, DHexUAa1–3GalNAc (4-O-sulfate); ΔDi-diSD, ΔHexUA(2-O-sulfate)a1–3GalNAc(6-O-sulfate); ΔDi-diSE, DHexUAa1–3GalNAc (4,6-O-disulfate). *t*-test with Welch’s correction; ***p < 0.001.﻿

Moreover, WFA staining is also associated with immunoreactivity of aggrecan, a major core protein of proteoglycans^32^. Aggrecan immunoreactivity was intense and PNN-like in the adult V1 of WT mice (Fig. 1F). T1 absence caused a significant reduction in aggrecan core protein (Fig. 1G), immunofluorescence intensity (Fig. 1I), and expression in V1 homogenates when quantified with immunoblotting (Fig. 1J). In contrast, the expression of other core proteins such as neurocan, phosphacan, brevican, and versican was not altered in T1 KO mice (Fig. 1J). Thus, genetic deletion of T1 not only caused a reduction in CS attached to multiple core proteins, but also specifically decreased the core protein aggrecan.

### Prevention of onset of the critical period by chronic CS reduction

**Figure 2:**
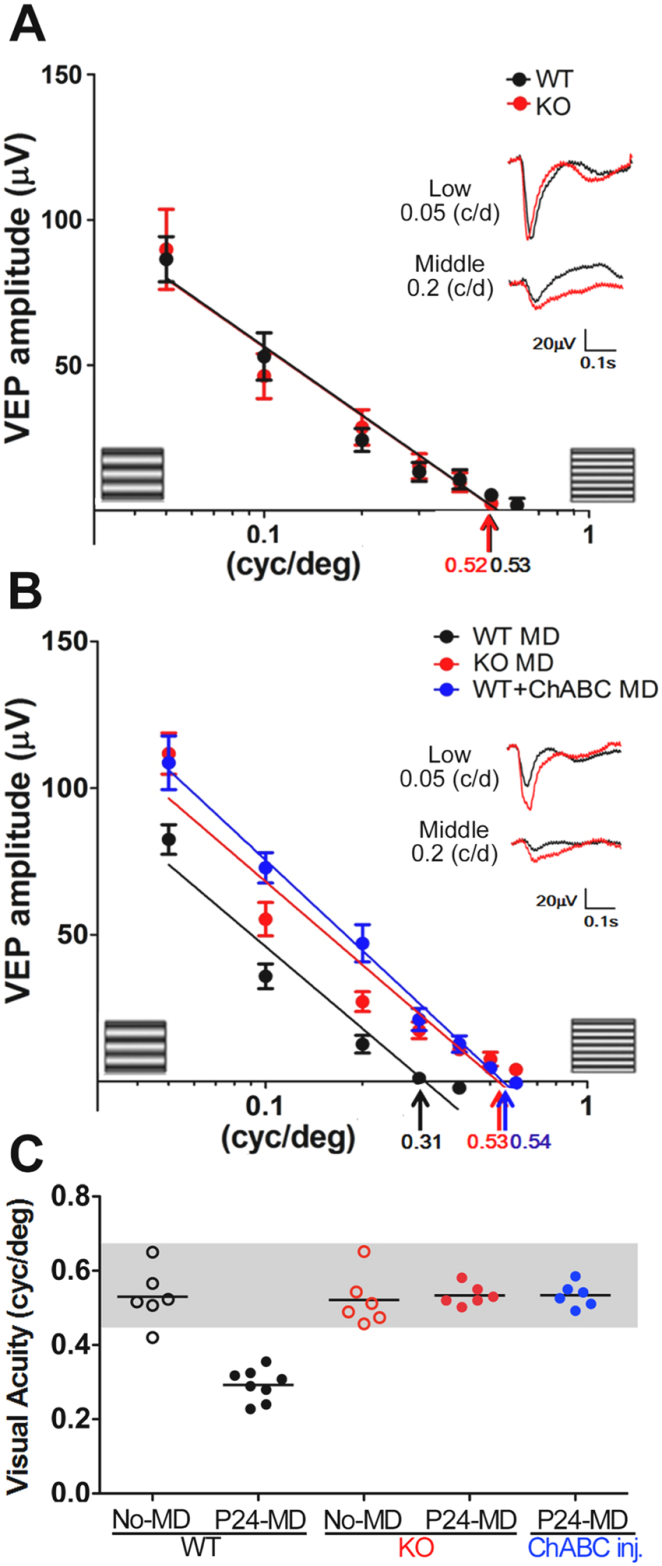
Endogenous CS reduction prevents deprivation-induced amblyopia. (**A**–**C**) VEP amplitudes of the first negative peak (mean ± SEM) and averaged traces (inset, for low or middle spatial frequency) reveal typical acuity of the non-deprived eye regardless of genotype (**A**, *black*, WT; *red*, KO). The acuity reduction by monocular deprivation (MD) during the critical period (*black*, WT) failed in T1 KO mice (*red*) or in mice treated with ChABC (*blue*) (**B**). Scatter plots of visual acuity for each mouse (**C**) without MD (No-MD, WT, 0.53 ± 0.03; KO, 0.52 ± 0.03) or with MD at P24 (P24-MD WT, 0.31 ± 0.02; KO, 0.53 ± 0.01; ChABC, 0.54 ± 0.01; MD versus No-MD, *p* = 0.0001 for WT, *p* = 0.98 for KO, *t*-test).

Because T1 deficiency caused a chronic reduction in CS during PNN formation, we next analyzed the functional role of CS in V1. First, we performed *in vivo* imaging of the endogenous fluorescence flavoprotein in response to visual stimuli. Neither the mean response amplitude nor the average position of the binocular zone, was significantly different between WT and KO mice (three mice, *p* > 0.27, *t*-test). Then, visual evoked potentials (VEPs) were recorded from the binocular zone of V1 in anesthetized mice (Fig. 2A, B). Visual acuity was estimated by extrapolating linear regression of the VEP amplitude versus the spatial frequency of the visual stimulus (range 0.05–0.7 cycles/degree) to 0 mV^33^. Juvenile (P28–30) T1 KO mice had an estimated spatial acuity of 0.52 cycles/degree, which was indistinguishable from age-matched WT controls (Fig. 2C, shaded area shows normal acuity^10,17^).

Then, we examined whether brief MD at P24 caused a rapid reduction in visual acuity (amblyopia). As expected, amblyopia of the deprived eye (0.31 cycles/degree) was induced in WT mice (Fig. 2B, C). In T1 KO mice, visual acuity remained normal and was not affected by brief MD. Similar results were obtained after removal of CS chains from WT visual cortices with chondroitinase ABC (ChABC) treatment accompanied by MD. Therefore, CS reduction by genetic deletion of T1 or enzymatic cleavage of CS, prevented the onset of plasticity and deprivation-induced amblyopia in juvenile mouse brains.

### Prevention of ocular dominance plasticity by chronic CS reduction

**Figure 3:**
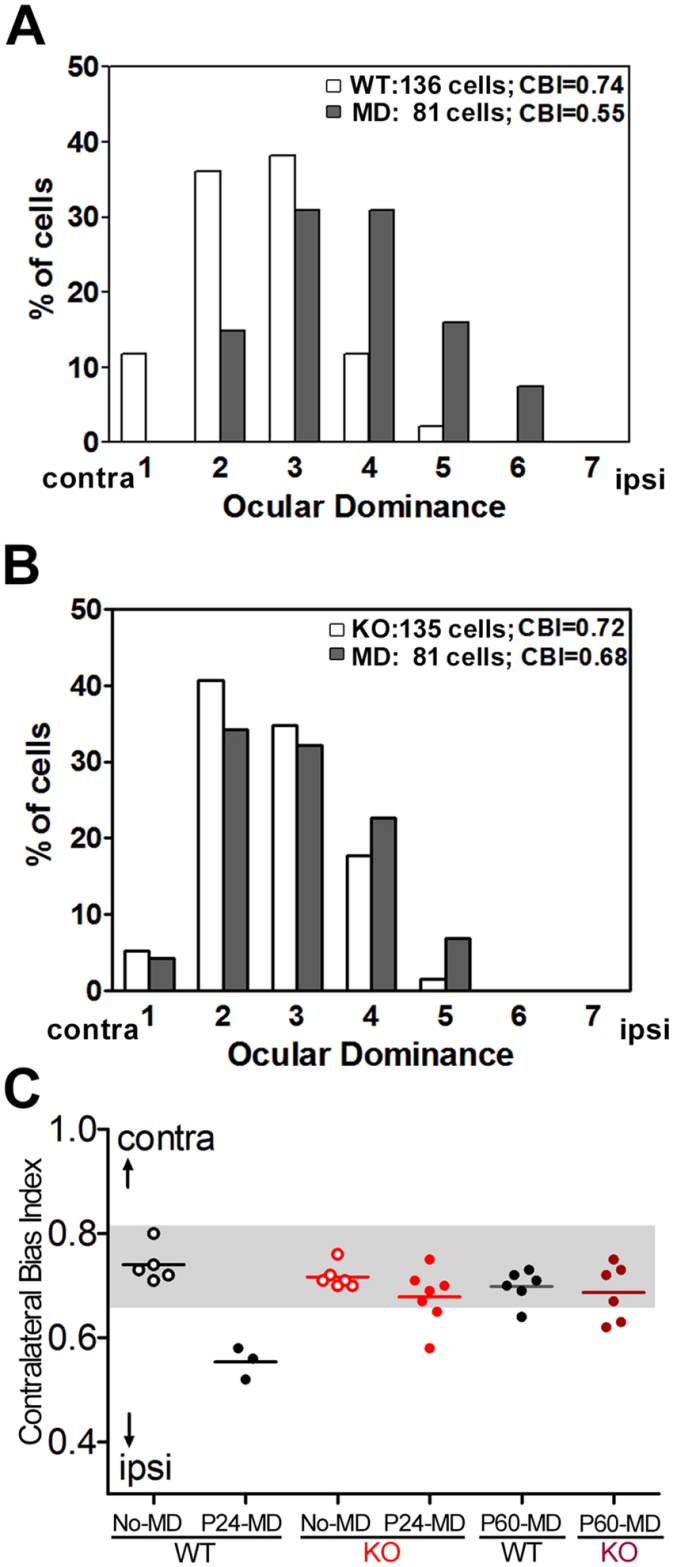
Endogenous CS reduction prevents ocular dominance plasticity. (**A**–**C**) Ocular dominance shift (toward group 4) is observed following MD in WT mice (**A**, No-MD, white bars; MD, gray bars; *p* < 0.0001, χ^2^-test), but is not induced in T1 KO mice (**B**, *p* > 0.05, χ^2^-test). High CBI values of each animal (**C**) typically shift following MD at P24 in WT mice (no-MD, 0.74 ± 0.02 versus P24-MD, 0.55 ± 0.02, *p* = 0.0003, *t*-test) but little shift is observed in T1 KO mice (no-MD, 0.72 ± 0.01 versus P24-MD, 0.69 ± 0.02, *p* = 0.14, *t*-test). Plasticity is not detected in adult WT (P60-MD, 0.70 ± 0.01) or T1 KO mice (P60-MD, 0.69 ± 0.02). contra, contralateral eye; ipsi, ipsilateral eye.

The spiking response to visual stimuli was also measured from the binocular zone with extracellular recording and was classified according to a traditional scale from group 1 (purely contralateral eye response) to group 7 (purely ipsilateral eye response)^2^. Unlike the contralaterally biased distribution in WT mice without MD (Fig. 3A), ocular dominance scores typically shifted toward the open, ipsilateral eye following a brief MD of the contralateral eye at P24 in WT mice, consistent with previous reports^2,4,11^. This reflected a reduction in the contralateral bias index (CBI) from 0.74 to the more balanced value of 0.55 (Fig. 3C, shaded area shows normal bias^2,4^). In contrast, T1 KO mice no longer showed this plasticity, as dominance of the deprived contralateral eye remained (Fig. 3B). The impaired plasticity in KO mice was clearly represented in a scatter plot of CBI values from individual animals (Fig. 3C). Similarly, ocular dominance plasticity was not detected again in adult KO mice (Fig. 3C), suggesting inactivation of the critical period throughout life. Thus, CS synthesis was essential to the normal activation of the critical period plasticity.

### Aggrecan promotes Otx2 uptake in juvenile mice via CS chains

**Figure 4:**
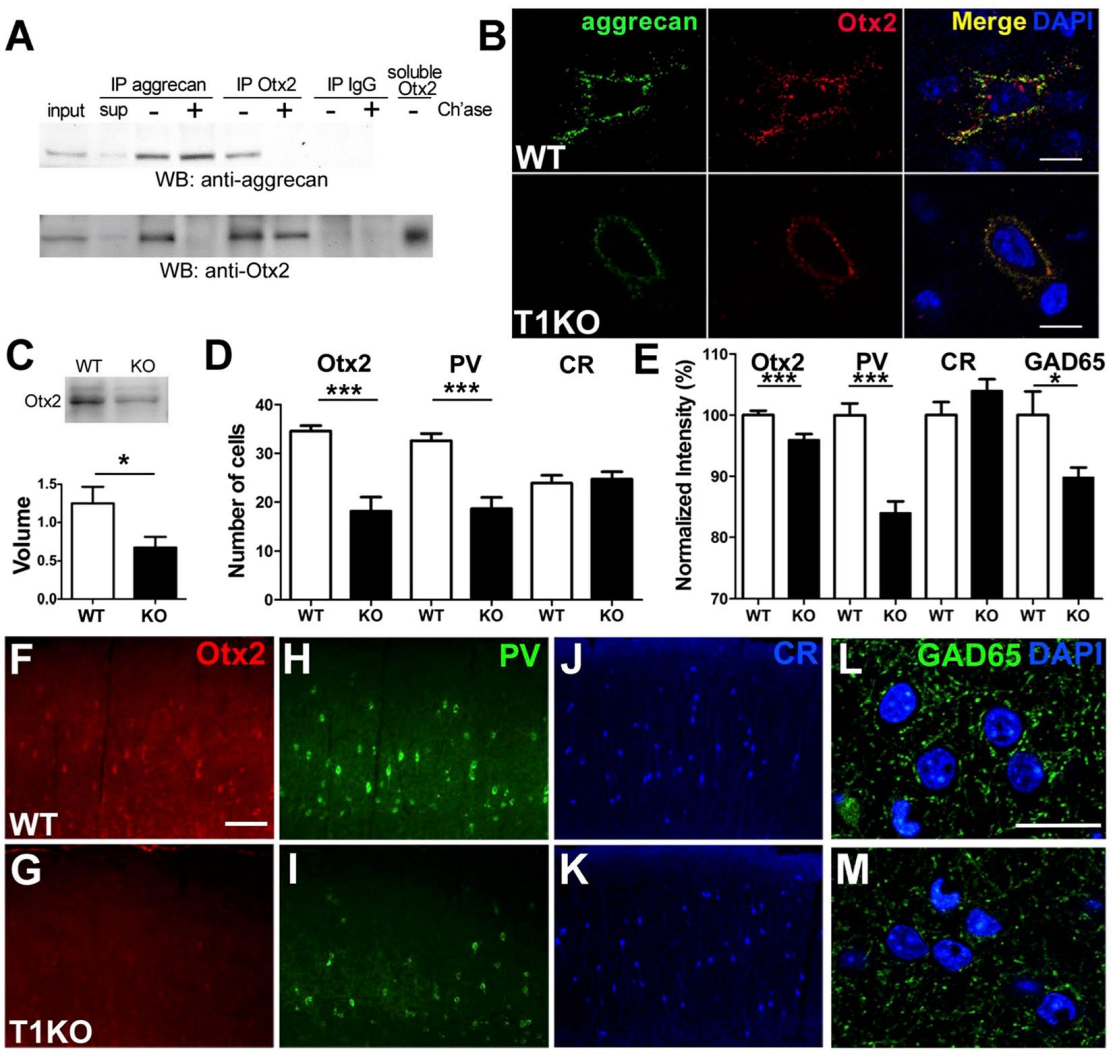
Inhibitory maturation requires aggrecan-dependent accumulation of Otx2 within PV-cells. (**A**) Co-immunoprecipitation (IP) of aggrecan and Otx2. Homogenates of V1 are treated with (+) or without (−) ChABC digestion (Ch’ase). Lanes from the left, input (1/40), supernatant (sup), IP with anti-aggrecan, anti-Otx2 antibody, normal IgG, soluble recombinant Otx2. The full-length blots are shown in a Supplementary Information. (**B**) Co-localization of Otx2 (red) with aggrecan (green) is observed in the binocular zone of the adult V1 with immunostaining using nonpermeabilizing conditions for labeling of extracellularly localized Otx2^23,24^ (top, WT; bottom, KO; right, merged images with DAPI). The scale bars represent 10 μm. (**C**) Reduction in Otx2 protein in juvenile (P28–30) T1 KO mice (top, specific bands for Otx2; bottom, relative volume of protein, six mice, *p* = 0.02, *t*-test). The full-length blots are shown in a Supplementary Information. (**D**) Decreased number of positive cells for Otx2 and PV in juvenile (P28–30) T1 KO mice, but no difference in the number of Calretinin (CR)-positive cells in supragranular layers of the binocular zone (600 × 350 μm; 4–6 mice on P28–30, WT 34.6 ± 1.1 versus KO 18.1 ± 2.9, *p* = 0.0007 for Otx2, WT 32.6 ± 1.46 versus KO 18.67 ± 2.29, *p* < 0.0001 for PV, WT 23.9 ± 1.6 versus KO 24.7 ± 1.5, *p* = 0.72 for CR, *t*-test). (**E**) Quantitative analysis of intensity within somata for Otx2, PV, and CR (600 × 350 μm; *p* < 0.0001 for Otx2 and PV, *p* > 0.05 for CR, *t*-test). Reduced intensity of GAD65 in supragranular layers of the binocular zone (400 × 350 μm; 3–4 mice, *p* = 0.038, *t*-test). (**F**–**M**) Reduction in Otx2 (**F**,**G**), PV (**H**,**I**), and GAD65 (**L**,**M**) staining in supragranular layers of the binocular zone in T1 KO (bottom) compared to WT (top) mice at P28–30. Notably, CR staining is not altered (**J**,**K**). The scale bars represent 100 μm (**F**–**K**) and 20 μm (**L**,**M**).

GAGs within PNNs have a strong affinity for Otx2, which activates cortical plasticity^11,23^. Thus, we analyzed whether Otx2 directly interacted with aggrecan in V1 or not. We performed co-immunoprecipitation with anti-Otx2 or anti-aggrecan antibody using protein lysates from V1 and detected Otx2 or aggrecan. Otx2 was co-immunoprecipitated with aggrecan, and vice versa (Fig. 4A). Pre-incubation of cortical lysates with ChABC blocked Otx2 binding to aggrecan, suggesting that Otx2 interacts with aggrecan-CSPG via its CS chains. Furthermore, double staining revealed co-localization of Otx2 and aggrecan in the dense PNN structure of V1 (Fig. 4B). Notably, aggrecan staining was more diffuse and weaker in T1 KO mice than in WT mice. Similarly, Otx2 accumulation in T1 KO mice was strikingly decreased at P28 (Fig. 4B–G) in the total amount (Fig. 4C), in the numbers of cells containing Otx2 (Fig. 4D: 4–5 mice, *p* = 0.0007, Welch’s *t*-test), and in the average immunofluorescence intensity within or around somata including the PNN structure (Fig. 4E: 346 cells from five WT mice, 145 cells from four KO mice, *p* = 0.0007, Welch’s *t*-test). Thus, CS synthesis was necessary for the interaction between Otx2 and aggrecan, and hence for Otx2 accumulation in PV-cells.

In agreement with a previous observation in Otx2 KO mice^11^, Otx2 removal by T1 deletion caused a significant reduction in the number of PV-cells (Fig. 4D, H, I: six mice, *p* < 0.0001, Welch’s *t*-test). The average intensity of PV immunostaining within the somata was decreased (Fig. 4E: 414 cells from six WT mice, 224 cells from six KO mice, *p* < 0.0001, Welch’s *t*-test). In contrast, neither the number of calretinin (CR)-positive cells nor their mean intensity was altered (Fig. 4D, J, K: five mice, *p* = 0.72, *t*-test; Fig. 4E: 239 cells from five WT mice, 247 cells from five KO mice, *p* = 0.16, *t*-test). Moreover, signals of GAD65-positive boutons surrounding somata were less intense and not compacted compared to control boutons (Fig. 4L, M). Quantitative analysis showed that the mean intensity of GAD65 was also decreased (Fig. 4E: 3–4 mice, *p* = 0.038, Welch’s *t*-test). Therefore, these results indicated that the inhibitory circuitry of PV-cells was weakened in T1 KO mice.

### Immature PV circuitry in the absence of sufficient CS

**Figure 5:**
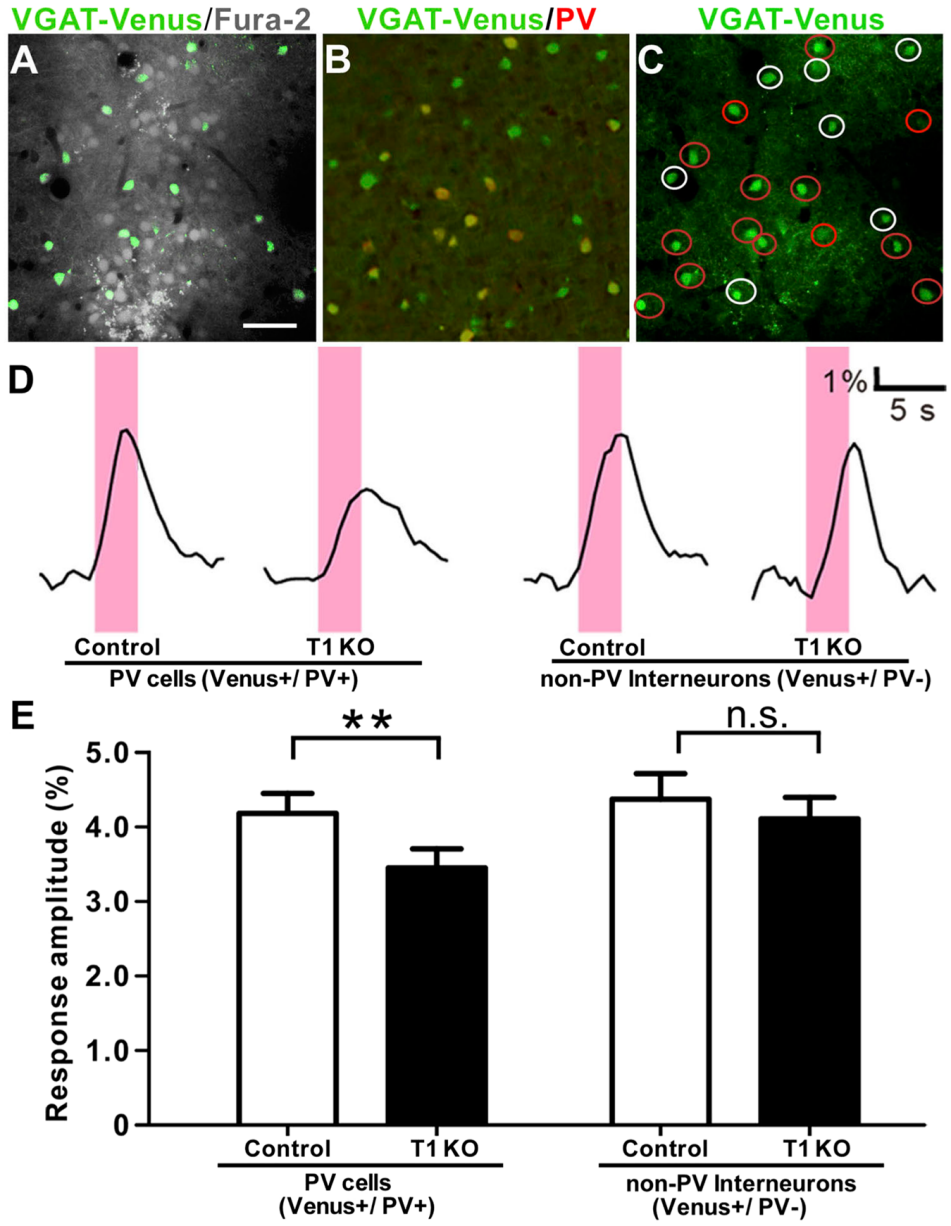
Two-photon imaging shows immature visual responses of PV-cells in T1 KO mice. (**A**–**C**) Identification of PV-cells or non-PV interneurons in two-photon imaging at P28–30. A recorded plane includes fura-2-positive (gray) and Venus-positive (green) neurons *in vivo* (**A**). PV-cells (red) revealed by immunostaining (**B**) are selected in a well-matched plane among *in vivo* images (**C**, *red circles*, PV-cells; *white circles*, non-PV interneurons). The scale bars represent 50 μm. (**D**) Visual-evoked fluorescence traces (ΔF/F0) from four neurons within the binocular zone. Pink bars indicate presentation of visual stimuli. (**E**) Significant reduction in response amplitudes (ΔF/F0, mean ± SEM) for PV-cells in T1 KO mice at P28–30 (63 neurons from three WT mice, 79 neurons from three KO mice, WT, 4.18 ± 0.26 versus KO, 3.44 ± 0.26, *p* = 0.002, *t*-test), but no effect in non-PV interneurons (45 neurons from three WT mice, 45 neurons from three KO mice, WT, 4.37 ± 0.34 versus KO, 4.10 ± 0.29, *p* = 0.59, *t*-test).

To analyze the functional effect of CS on PV circuitry, we recorded visual responses from the binocular zone using two-photon calcium imaging. We measured Ca^2+^ signals from interneurons that expressed the fluorescent protein Venus via the vesicular GABA transporter (VGAT) promoter, with rapid injection of fura2, a Ca^2+^ indicator with a distinct excitation wavelength from Venus fluorescence^34^ (Fig. 5A). Post-hoc immunostaining following two-photon imaging allowed us to distinguish PV from non-PV cells among the recorded interneurons (Fig. 5B, C). Analysis of calcium transients in response to visual stimuli revealed that the mean amplitude for PV-cells was significantly decreased by T1 deletion compared to control (Fig. 5D, E: three mice, *p* = 0.002, *t*-test). Conversely, the amplitude in non-PV interneurons was indistinguishable from the control response (three mice, *p* = 0.59, *t*-test). Thus, not only was the number of PV-cells reduced by nearly half (Fig. 4), but the function of the remaining PV circuitry was also strikingly weakened in T1 KO mice. Moreover, T1 deletion also impacted the amplitude in non-GABAergic pyramidal neurons (281 neurons from three WT mice, 391 neurons from three KO mice, WT, 3.79 ± 0.11 versus KO, 3.27 ± 0.09, *p* < 0.05, *t*-test), suggesting that the lack of T1 reduced CS around pyramidal neurons (Fig. 1).

### Excess Otx2 restores cortical plasticity

**Figure 6:**
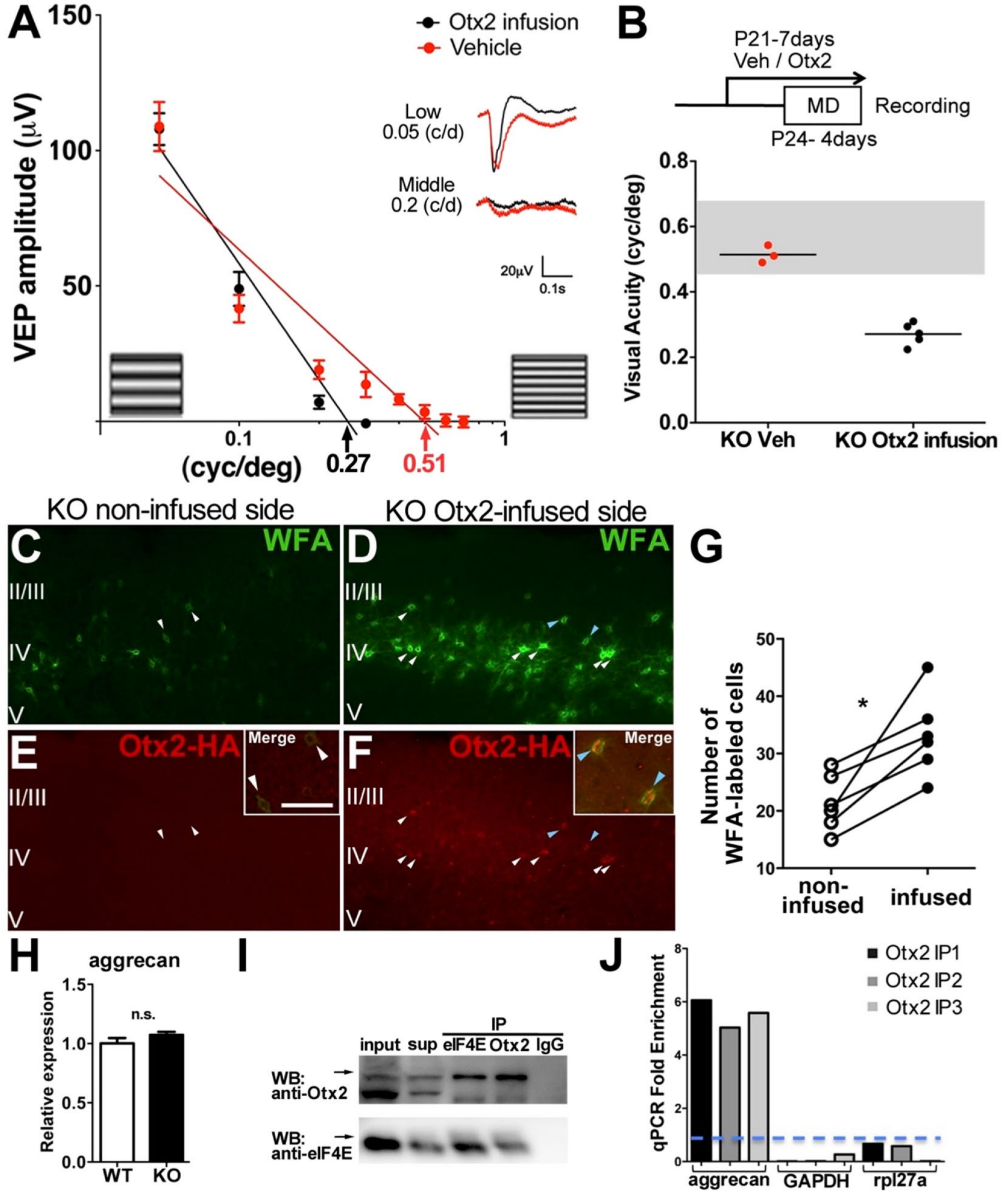
Otx2 infusion restores the plasticity and expression of CSPG. (**A**) VEP amplitudes of the first negative peak (mean ± SEM) and averaged traces (inset, for low or middle spatial frequency) reveal the acuity of the deprived eye in Otx2-infused T1 KO (*black*) or vehicle-infused KO mice (*red*). (**B**) Visual acuity of individual T1 KO mice (*bottom*) infused from P21 to P28 and deprived at P24 (*top*). Otx2-infused mice showed a significant acuity reduction in the deprived eye (*black symbols*, 0.27 ± 0.02 cycles/degree) compared to vehicle-infused KO (*red symbols*, 0.51 ± 0.02 cycles/degree, *p* < 0.0001, *t*-test). (**C**–**F**) Infused Otx2 (red) facilitates WFA staining (green) in T1 KO mice (**D**,**F**) compared to the opposite non-infused hemisphere in the same brains (**C**,**E**). Otx2-HA is internalized in WFA-labeled cells (inset in **F** compared to in **E**). The scale bars represent 100 μm. (**G**) Numbers of WFA-labeled cells are increased in Otx2-infused T1 KO mice (600 × 350 μm; numbers of cells, non-infused side 21.7 ± 1.05 versus Otx2-infused side 33.8 ± 2.3, six mice, *p* = 0.003, paired *t*-test). (**H**) Normalized mRNA expression of *aggrecan* in juvenile V1. Note that despite a specific decrease in aggrecan protein, the transcripts detected with RT-qPCR were not reduced in T1 KO (six mice, *p* > 0.05, *t*-test). Error bars represent SEM. (**I**) Co-immunoprecipitation (IP) of eIF4E and Otx2. Lanes from the left, input (1/40), supernatant (sup), IP with anti-eIF4E, anti-Otx2 antibody, normal IgG. The full-length blot in a Supplementary Information. (**J**) Association between Otx2 and *aggrecan* mRNA in juvenile V1. Three independent Otx2 immunoprecipitates and their initial input samples were analyzed for aggrecan, rpl271, and GAPDH mRNAs. Relative fold enrichment of each mRNAs is shown and compared to the input (hatched blue line).

Given that impaired plasticity by T1 deletion is due to the absence of Otx2, we attempted to activate the critical period by administering exogenous Otx2. HA-tagged Otx2 protein or vehicle was directly infused into one hemisphere of V1 in T1 KO mice with an osmotic minipump beginning on P21^11^. Vehicle treatment had no effect on visual acuity of the eye deprived at P24 (Fig. 6A, B). In contrast, spatial acuity of the deprived eye was reduced with Otx2 infusion, and an amblyopic effect was clearly observed in individual animals in scatter plots (3–5 mice, *p* < 0.0001, *t*-test). Staining for the HA-tag revealed that exogenous Otx2 was preferentially internalized into WFA-labeled cells on the infused side compared to the non-infused side in the same brain (Fig. 6C–F). Surprisingly, after 7 days of infusion, a robust increase of WFA staining was observed in the Otx2-infused cells (Fig. 6G: six mice, *p* = 0.003, paired *t*-test). Hence, excess Otx2 activated plasticity in T1 KO mice, indicating that Otx2 acted as a downstream factor of CS.

To examine whether Otx2 is involved in transcriptional regulation of aggrecan, we performed quantitative RT-PCR with V1 homogenates. Transcripts of aggrecan were not affected in T1 KO (Fig. 6H) or Otx2 KO mice (6–9 mice, *p* > 0.05, *t*-test) in which the number of WFA-labeled cells was also reduced^11^, suggesting that Otx2 was not directly involved in transcriptional activation of *aggrecan*. Importantly, some homeoproteins may be associated with the translational machinery in embryos^35,36^. Indeed, co-immunoprecipitation with anti-Otx2 or anti-eIF4E antibody revealed that Otx2 was immunoprecipitated with the translation initiation factor eIF4E from V1 lysates (P28–30), and vice versa (Fig. 6I). In addition, quantitative RT-PCR in three independent immunoprecipitates each with anti-Otx2 antibody and nonspecific IgG showed that Otx2 was associated with a subset of mRNA (Fig. 6J). *Aggrecan* mRNA was detected in the immunoprecipitates with Otx2 (more than 5.0-fold enrichment), but not in those with rabbit IgG (less than 1.6-fold enrichment). In contrast, transcripts of the house-keeping gene, rpl27a or GAPDH were not enriched in the Otx2 or IgG immunoprecipitates, suggesting that the association between Otx2 and *aggrecan* mRNA is specific. Thus, aggrecan expression may be post-transcriptionally promoted by Otx2 in association with the eIF4E complex.

### DZ triggers persistent plasticity in the absence of CS or Otx2

**Figure 7:**
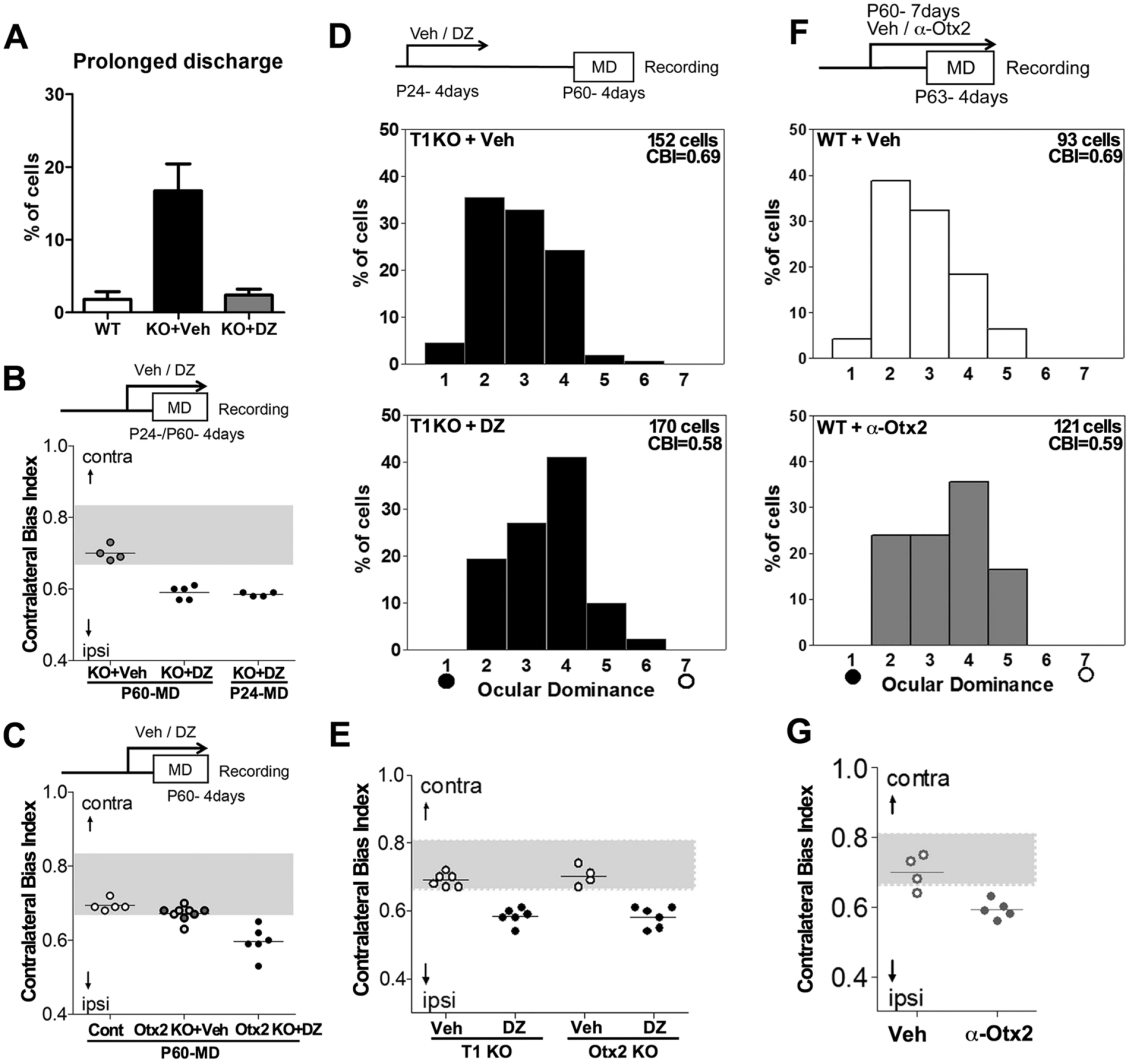
The offset of visual plasticity is also prevented in both T1 and Otx2 KO mice. (**A**) Prolonged neuronal firing is elevated in T1 KO mice (KO + Veh) and reduced by DZ treatment (KO + DZ) as well as WT mice (10–16 mice, WT 1.8 ± 1.0 versus vehicle-injected KO 16.7 ± 3.7, *p* < 0.0001, DZ-injected KO 2.4 ± 0.8 versus vehicle-injected KO, *p* < 0.0001, ANOVA). Error bar represents SEM. (**B**) CBI values of vehicle-injected T1 KO mice are typically high with MD over P60 (KO + Veh, 0.70 ± 0.01). Reduction in CBI values is induced in DZ-treated KO with MD over P60 (+DZ, 0.59 ± 0.01, versus + Veh, *p* < 0.0001, *t*-test) as well as at P24 (+DZ, 0.59 ± 0.002). (**C**) High CBI values in the deprived control (Cont, 0.69 ± 0.01) and Otx2 KO mice (KO + Veh, 0.67 ± 0.01; *open symbols*, non-injected; *gray symbols*, vehicle-injected) are significantly reduced by DZ injection (+DZ, 0.59 ± 0.02) even in the adult (>P60) (KO + Veh versus + DZ, *p* = 0.0003, *t*-test). (**D**) Prolonged critical period in T1 KO mice. Ocular dominance shift following MD (toward group 4) is observed even 1 month later after DZ at P24 (T1 KO + DZ), but not after vehicle injection (T1 KO + Veh) (*p* < 0.0001, χ^2^-test). (**E**) High CBI values in deprived T1 KO (0.69 ± 0.01) or Otx2 KO mice (0.70 ± 0.01, Veh, vehicle-injected mice) are reduced long after the onset in DZ-injected mice (DZ, T1 KO, 0.58 ± 0.01 versus Veh, *p* < 0.0001; Otx2 KO, 0.58 ± 0.01 versus Veh, *p* = 0.0003, *t*-test). (**F**) Otx2 removal by cortical infusion of an inhibitory antibody (α-Otx2) reactivates plasticity in adult WT mice (WT + Veh, vehicle-infused; WT + α-Otx2, antibody-infused; *p* < 0.0001, χ^2^-test). (**G**) Ocular dominance shift (low CBI) in individual mice is induced by acute inhibitory antibody (α-Otx2) with MD (Veh, 0.70 ± 0.02 versus α-Otx2, 0.59 ± 0.01, *p* = 0.004, *t*-test).

In younger WT mice before the critical period, prolonged spike firing is typical after a visual stimulus in the receptive fields has ended (prolonged discharge). Prolonged discharge reflects weak inhibition *in vivo* ^5^. Consistent with immature PV circuitry with T1 deletion (Fig. 4), prolonged discharge was observed in a significantly higher number of T1 KO cells as observed with extracellular recording from the binocular zone (Fig. 7A). As expected, the ratio was reduced with 4 days of DZ treatment, which enhances GABA_A_ receptor-mediated currents (10–16 mice, *p* < 0.0001, ANOVA).

We then examined whether enhancing inhibition could restore the plasticity at any time throughout life. Ocular dominance plasticity was not induced again in vehicle-injected T1 KO mice (>P60; Fig. 7B), similar to adult WT or KO mice (Fig. 3C). Importantly, the CBI values decreased toward the open ipsilateral eye following deprivation of the contralateral eye on P24 or P60 with DZ treatment. Thus, plasticity was activated any time by DZ, even in adulthood as well as juvenile mice. Similarly, in adult controls and Otx2 KO mice, ocular dominance shifts were not detected following MD, but were activated with DZ treatment in Otx2 KO mice (Fig. 7C). Therefore, insufficient CS or Otx2 retained PV circuitry in the pre-critical period state, and consequently, enhancing this circuit was sufficient to activate the onset of plasticity throughout life. WFA staining showed no significant increase following DZ treatment (vehicle-injected KO mice 32.1 ± 1.7 versus DZ-injected KO mice 32.4 ± 1.5, eight mice, *p* = 0.87, *t*-test), confirming that this drug functions through GABA_A_ receptors at postsynaptic sites in pyramidal neurons and interneurons.

Once the critical period is induced, plasticity declines within 2 weeks of onset in normal mice^3^. CS enrichment acts as a brake for excess plasticity in the adult V1 and spinal cord^21,37^. Interestingly, we found that after plasticity was triggered by DZ treatment (from P24 to P27) in T1 KO mice (Fig. 7B), the critical period was sustained even in adulthood (>P60; Fig. 7D, E). Extracellular recording revealed that ocular dominance shifted toward the open ipsilateral eye with MD, even 1 month after DZ, but not after vehicle treatment. Similarly, persistent plasticity was observed in adult Otx2 KO mice after the onset was triggered with DZ treatment (Fig. 7E).

To further confirm whether endogenous accumulation of Otx2 restricts plasticity as well as CS^21^, we prevented Otx2 uptake in the adult V1 with cortical infusion of an inhibitory antibody for Otx2 (α-Otx2) using an osmotic minipump^11^. Ocular dominance plasticity was reactivated by removal of Otx2 even in adulthood (>P60; Fig. 7F, G), consistent with our previous result^23^. The relationship between CS and Otx2 was reciprocal, and acute removal of Otx2 reduced the CS content^23^, hence reverting an inflexible or mature PV circuit to a plastic state. Thus, our results confirmed that endogenous accumulation of either CS or Otx2 restricts the critical period plasticity after the onset.

### Developmental state of the PV circuitry regulated the window of the critical period

**Figure 8:**
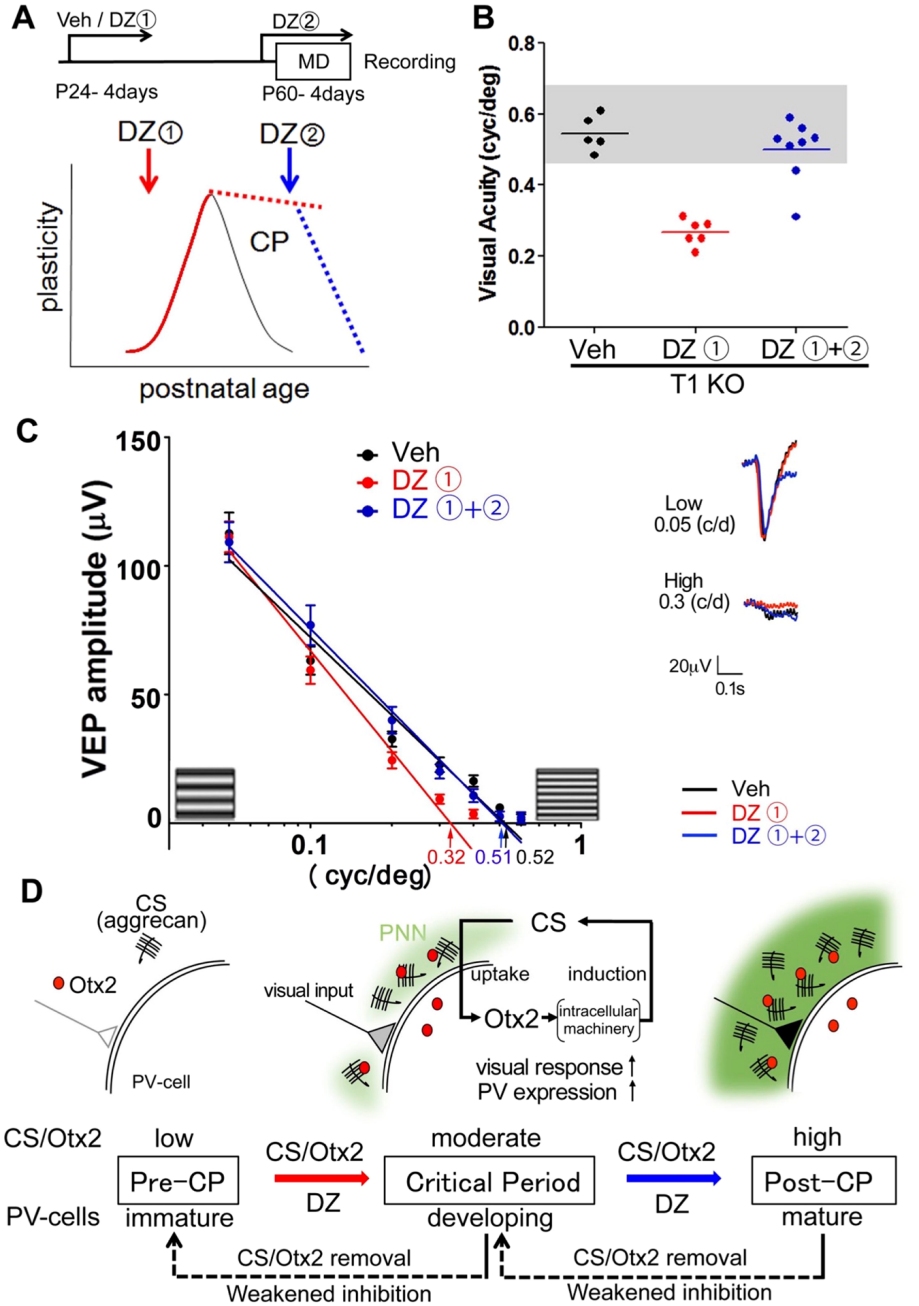
Critical period plasticity according to the developmental stage of PV-cells. (**A**) Proposed bidirectional role of inhibitory circuits at the onset (*red*) and offset (*blue*) of the plasticity. (**B**) First, DZ activates persistent plasticity (low acuity of the deprived eye, *red symbols*) in T1 KO mice (DZ1, 0.29 ± 0.01 versus Veh, 0.54 ± 0.02, *p* < 0.0001, ANOVA). Second, DZ restricts the plasticity (high acuity of the deprived eye, *blue symbols*) (DZ2, 0.50 ± 0.03 versus DZ1, *p* < 0.0001, ANOVA). (**C**) VEP amplitudes of the first negative peak (*left*, mean ± SEM) and averaged VEP traces (*right*, for low or high spatial frequency) reveal an amblyopic effect on the deprived eye long after the first DZ injection from P24 in T1 KO mice (*red line*), and conversely, no effect after vehicle (Veh, *black line*) or additional DZ injection from P60 (*blue line*). (**D**) A schematic model for CS-Otx2 accumulation and PV-cell maturation. Initial emergence of CS and Otx2 (*red arrow*) promotes the onset of the critical period (*CP*), and then further accumulation (*blue arrow*) leads to the offset. In both steps, the interaction between CS and Otx2 is crucial for PV-cell function, which is substituted by DZ treatment. CS-attached aggrecan in PNNs (*green*) increases Otx2 uptake, and internalized Otx2 further promotes aggrecan expression in PV-cells (*cartoon*). This positive loop enhances the visual response and PV expression from the beginning of their weak properties and is finally involved in the inflexibility of this local circuitry (at post-CP). The plastic state (*center*) can be activated or inactivated, namely *bidirectionally*; for example, artificial treatment such as acute removal of CS or Otx2 (*hatched black arrow*) resets CP (plastic state) to pre-CP (immature state), and the same applies to post-CP (mature state) that is reset to CP (plastic state).

The CS-Otx2 interaction was involved in restricting plasticity, as well as activating plasticity. To address whether PV circuitry is further implicated in declining plasticity, we applied additional DZ (from >P60) following the first treatment (from P24) in T1 KO mice (Fig. 8A). VEPs were recorded from the binocular zone. Visual acuity was not affected by MD with vehicle injection (Fig. 8B, C). Amblyopia of the deprived eye was again detected 1 month after the first DZ treatment, confirming that once the immature PV circuitry was enhanced at a younger age, the plasticity was retained in adulthood (Fig. 7). Importantly, T1 KO mice given additional exposure to DZ no longer showed the amblyopic effect in the deprived eye (Fig. 8B, C). The long-lasting plasticity declined with the second DZ treatment, which further enhanced the inhibition that was implicated in the PV circuitry. Taken together, the functional state of PV-cells that is regulated by the CS-Otx2 interaction or by DZ may modulate the critical period plasticity (Fig. 8D).

## Discussion

Our results demonstrated that CS, which acts as a molecular “brake” in plasticity, also functions as an inducer of plasticity (Fig. 8D). The bidirectional effect of CS may be dose-dependent; the initial presence of CS promotes the onset of the critical period, and further accumulation induces its offset. As the CS amount is raised by Otx2, the function of PV-cells is enhanced. We therefore propose a common molecular mechanism between onset and offset of plasticity.

CSPGs in PNNs are first observed in the postnatal 2 weeks^21,30^, together with other PNN components such as hyaluronan and the link protein^22^. Regarding CS biosynthesis, T1 expression also persists before the critical period and is required to optimize the CS content surrounding PV-cells in the juvenile V1 (Fig. 1). T1 is believed to regulate the rate-limiting step of CS synthesis *in vitro* among more than 10 species of CS-synthesizing enzymes^27^. T1 inactivation not only reduced the total amount of CS, but also the specific CSPG aggrecan, which has more CS chains than other CSPGs, indicating the indispensable role of this enzyme *in vivo*. Our study first observed that inactivation of a single enzyme, T1, results in impaired visual responses of PV-cells, hence inhibiting the onset of the critical period (Figs 2–5). These results demonstrate the direct contribution of endogenous CS to the activation of plasticity.

CS chains are considered molecular reservoirs of diffusible factors and the homeoprotein Otx2^38^. Otx2 contains an RK motif, which is a 15-aa peptide (RKQRRERTTFTRAQL) corresponding to a putative GAG-binding motif^23^. In agreement with previous studies using adult animals, cortical Otx2 was strongly associated with CS chains of aggrecan in PNNs (Fig. 4). Even in juvenile animals, CS depletion prevented Otx2 uptake into PV-cells and hence, plasticity, which was restored by Otx2 infusion (Fig. 6). These observations indicate that CS emergence during the critical period plays an essential role in capturing endogenous Otx2.

Under the deprived conditions like dark rearing, the maturation of PNNs is delayed in V1, accompanying with reduction of CSPG core proteins and Otx2^21,22,30^. Our results showed that Otx2 infusion restored the expression of aggrecan and hence promoted the PNN formation even in darkness^11^ or T1 deletion (Fig. 6), suggesting that the accumulation of aggrecan induced by Otx2 is important to PNN formation. In contrast, the fact that PNNs structure is still immature even in the slightly-increased T1 expression in dark rearing^11,21,22^ (around 1.2 times, Fig. 1) suggested that the enzymatic activity of T1 solely was not enough to promote the maturation of PNNs. The roles for other components in PNNs for the maturation of PNNs should be further elucidated.

As CS accumulates, the net negative charges in PNNs should increase. In addition, CS accumulation is accompanied by a growing diversity in CS sulfation patterns, which are classified into five dissaccharide structures by the position of sulfation^22,24^ (see Table 1). Therefore, a quantitative increase in CS chains, which leads to qualitative changes of PNNs, may distinguish juvenile CS from adult CS because CS interacts with a number of molecules via its negative charges. Molecules that induce the onset of plasticity such as BDNF^10,39^ and Narp^12,40^ as well as Otx2 bind to juvenile CS. In the mature PNN, for example, a report has shown that CS is a ligand for NgR1^41^, which functions in the offset of plasticity in PV-cells^42^. Thus, juvenile CS may recognize factors that initiate the critical period, and adult CS may bind inhibitory factors that restrict plasticity.

In addition to CS enrichment in PNNs, CS spreads in the extracellular matrix around pyramidal neurons and affects the structural and functional plasticity of their dendritic spines^43,44^. Indeed, remodeling of dendritic spines, which is concomitant with ocular dominance plasticity, is induced by tissue plasminogen activator (tPA) to digest extracellular matrix^45–47^. Moreover, maturation and unsilencing of excitatory synapses via postsynaptic density protein −95 (PSD-95) is required for closing the critical period but not for opening it^48,49^. Interestingly, inhibitory circuits seem to be intact in tPA or PSD-95 KO mice so that DZ cannot restore the timing of the critical period, indicating that excitatory function plays an indispensable role for the plasticity^45,49^. Our data showed that visual responses in pyramidal neurons were also reduced in T1 KO mice, suggesting an impact of CS reduction on excitatory circuits. Although unlike tPA or PSD-95 KO mice, DZ could restore the onset and offset of the critical period in T1 deletion and thus weakened inhibition in CS reduction was involved in the plasticity, the contribution of CS around pyramidal neurons could not be ruled out. It would be important to examine whether CS-Otx2 interaction contributes the maturation of excitatory circuits in future studies. Alternatively, the recent findings shed light on the fact that some environment can induce the juvenile-like plasticity in adult mice via modifying inhibitory circuits or PNNs^13,19,50,51^. More conceptually, a possibility that restricted plasticity in the adult mice raised in the impoverished condition like standard laboratory cages may suggest a future issue to make raising environment more natural and realistic in order to find out the ‘real’ molecular mechanisms and therapeutic strategies for human amblyopia.

Our results demonstrated the immature visual response of PV-cells in T1 KO mice using two-photon imaging (Fig. 5) and impaired plasticity in both T1 KO and Otx2 KO mice, which were restored by DZ (Figs 7 and 8). These findings indicate that the GABAergic effects derived from PV-cells require both CS and Otx2.

The dosage effect of CS was entirely consistent with recent findings in the field of plasticity. Compared to our results, mice with a mild reduction in CS due to the disruption of Crtl1 or overexpression of chondroitin 6-sulfotransferase show no change in the number of PV-cells or initiation of the critical period^22,24^. Importantly, these mice only show a failure to terminate plasticity, suggesting that a full dosage of CS acts as a physical barrier in synaptic plasticity^44^. Meanwhile, our study with direct deletion of a key CS-synthesizing enzyme revealed that severely reduced CS (~59%) is not sufficient for initiation or termination of the critical period, concomitant with almost half the number of PV-cells. PV expression is closely correlated with PV-cell activity, according to a reduction in expression in sensory deprivation or in the pre-critical period when PV-cell responses are immature^9,11,52^. Indeed, in our two-photon imaging analysis, reduced CS weakened visual responses of the remaining PV-cells, which can further decrease PV expression. Therefore, CS may contribute to development of PV expression and networks, perhaps through promoting visually evoked responses on PV-cells.

Optimizing the cortical PV networks is essential to postnatal plasticity across brain regions^53^. Too low or too high PV expression (and function) inhibits developmental plasticity for establishment of visual function^54^ and for multisensory integration in the insular cortex^55^ and impedes plasticity in hippocampal or fear learning^56,57^. Our study revealed that the CS-Otx2 interaction mediates postnatal maturation of PV-cells and maintenance throughout adulthood, indicating that the critical period is activated during PV-cell maturation when PV networks are optimal. Similarly, a brief DZ treatment optimizes the number of GABA_A_ receptors on a somatic region of pyramidal cells^7^ and this effect is persistent even 2 months later^58^. Unlike an agonist of GABA receptors such as pentobarbital anesthetics, the action of DZ is known to be dependent on the intrinsic GABA so that can selectively modulate the somatic inhibition with the long-lasting effect^7,58^. Therefore, a chronic DZ treatment might be capable of substituting CS-Otx2 system for the critical period.

During the critical period, MD drives rapid modulation of PV-cell activity to enhance visual circuitry from the non-deprived eye^8,9^. Importantly, translational control within PV-cells is involved in activity-dependent expression of genes that tune PV-cell responses^59^. Otx2 includes a putative eIF4E-binding motif (YxxxxLϕ, where ϕ is any hydrophobic), and homeoproteins are associated with eIF4E complexes to promote protein synthesis^35,60^. Our present results suggest that such an action of Otx2 upregulates aggrecan expression in PV-cells, consequently enhancing its responses. Note that rapid ocular dominance plasticity requires protein synthesis in cortical cells^61^. Otx2 may promote the protein synthesis for plasticity.

We showed that the CS-Otx2 interaction induces not only the termination but also the initiation of plasticity (summarized in Fig. 8D). If two distinct processes share a common molecular basis, what mechanism explains this regulation ? One possible explanation is that the Otx2-dependent increase in the amount of CS in PNNs regulates the critical period via PV-cell development. Indeed, we demonstrated that two genetic mutants lacking T1 and Otx2 required DZ administration twice to induce the entire phase of the critical period. The first application was necessary to initiate a plastic state with optimized PV circuitry, and the second one was needed to end it with highly activated PV-cells (Fig. 8). In contrast, only a single treatment is effective for restoring the entire phase of the critical period in mice with intact CS-Otx2 interactions, but the inhibition is not sufficient for either the onset^5^ or offset^17,42^ of plasticity. Taken together, the positive regulatory loop between CS accumulation and Otx2 uptake may act as a molecular timer to measure the timing of onset and offset of the critical period.

Finally, some human hereditary neuropsychiatric diseases are caused by mutations in T1 or Otx2^62,63^. Growing evidence proposes that disrupted PV networks in developing brain circuitry are relevant to neuropsychiatric conditions^54,55,64^. Our results suggest a therapeutic strategy for optimizing GABA function and a potential for CS chains for identifying the etiology of these conditions.

## Materials and Methods

### Animals

Animal experiments were performed in accordance with the experimental protocol approved by the Committee for Animal Care at Niigata University (Ref. No. 27-SHINDAIKENDAI39-3). All mice are housed in groups of 2–3 together with the sibling of the same sex in the standard and uniform cage sizes (143 × 293 × 148 mm, Charles river) and maintained on a 12 h light/dark cycle with *ad libitum* access to food and water. After preceding procedure like MD or drug infusion (see below), mice were returned to their home cages until next experiments. Conventionally raised C57Bl/6 J mice of both sexes were purchased from Japan SLC. T1 KO mice of both sexes were bred, genotyped, and maintained as described previously^31^. To visualize GABAergic neurons, T1 KO mice were mated with VGAT-Venus mice^65^. The Otx2 flox mice^66^ were crossed with CaMKII-Cre mice^67^ to generate mice of either sex with conditional Otx2 KO in the postnatal brain^11^.

### Quantitative morphological analysis

For *in situ* hybridization, deeply anesthetized mice were perfused transcardially and post-fixed with 4% paraformaldehyde. Floating coronal sections (20 μm) were hybridized at 65 °C with a digoxigenin-labeled RNA probe for T1^31^, and the probe was then detected with alkaline phosphate-conjugated anti-digoxigenin antibody^11^. For immunohistochemistry, floating coronal sections (20 μm) were incubated with biotin-labeled lectin from WFA (Sigma) or the following primary antibodies: anti-PV (Swant), anti-Otx2 (Millipore, Santa Cruz), anti-calretinin (Millipore), anti-GAD65 (Millipore), anti-aggrecan (Millipore), anti-HA (Roche). Alexa-conjugated anti-mouse, anti-rabbit, anti-goat, or streptavidin were used for secondary detection (Invitrogen).

Quantitative analysis of fluorescence intensity was performed as described previously^11^. Immunostaining for experimental control and sample sections was performed concurrently with the same solutions, and images were photographed in one sitting with the same gain and exposure time. The number of cells or average fluorescence intensity for each cell was measured with the spot module of NIS-Elements AR Analysis software (Nikon) in a 600 × 350 μm area covering the supragranular layers of the binocular zone. WFA + /Otx2 + /PV + /CR + cells were defined by combining threshold (between the intensity values of 328 to 4096) and area size (above 87 μm^2^) to distinguish positive cells from background signal. The number of positive cells was compared pair-wise across the same brain (paired *t*-test), or unpaired between two groups of animals (Welch’s *t*-test or Student’s *t*-test) or among groups (one-way ANOVA). For quantification of average fluorescence intensity of GAD65 or binarized images of CSPGs, we measured the areas in the supragranular layers of the binocular zone (450 × 350 μm) using NIS-Elements AR Analysis software (Nikon) or ImageJ software (NIH), respectively, and compared the data between two groups (Welch’s *t*-test or Student’s *t*-test).

### Biochemical analysis

Extraction of CS was performed as described previously^24^. The acetone powder from V1 regions was completely digested with actinase E (Kaken Pharma) and treated with 5% trichloroacetic acid (wt/vol). The acidsoluble fractions were extracted with diethyl ether, and the aqueous phase was neutralized and subjected to gel filtration on a PD10 column (GE Healthcare). An aliquot of the sample was digested with ChABC (Seikagaku), derivatized with a fluorophore, 2aminobenzamide, and then analyzed with anionexchange highperformance liquid chromatography (SLC10 A, Shimadzu) on a PA03 column (YMC).

Immunoblotting and immunoprecipitation were performed as described previously^68^. For immunoblotting of CSPGs, V1 lysates were digested with ChABC (50 mU/ml), and equal amounts of protein samples (40 μg) were electrophoresed. The following primary antibodies were used: anti-neurocan (R&D), anti-aggrecan (Millipore), anti-brevican (BD Biosciences), anti-phosphacan (DSHB), anti-versican (Millipore), and anti-Tubulin 4α (GeneTex) for normalization. The relative volume between a given sample and a reference was calculated with ImageLab software (Bio-Rad). For immunoprecipitation, Protein Sepharose 4 Fast Flow (GE Healthcare) or Dynabeads protein A beads (Invitrogen) were conjugated to 2.5 μg anti-Otx2, anti-aggrecan, or anti-eIF4E (Cell Signaling Technology) antibody or normal rabbit IgG (Santa Cruz). V1 lysates were treated with or without ChABC and incubated with Protein A beads. After washing and equilibrating, precipitated proteins were eluted by addition of elution buffer (pH 2.7, 50 mM Glycine-HCl) or boiling. Precipitated proteins were analyzed with immunoblotting.

RNA immunoprecipitation was performed with the RNA immunoprecipitation Assay Kit according to the manufacturer’s instructions (MBL). V1 lysates were precleared and incubated with Protein A beads conjugated to anti-Otx2 antibody (Millipore) or normal rabbit IgG (MBL). The quality of extracted total RNA (input) or immunoprecipitated RNA (IP) was analyzed on a BioAnalyzer (Agilent). RNA samples were reverse transcribed using the PrimeScript II kit (Takara), followed by quantitative reverse-transcription PCR.

### Quantitative real-time PCR

For expression analysis, extraction of total RNA from the V1 area and first strand cDNA synthesis were performed according to publisher recommended protocols with NucleoSpin RNA II reagent (Macherey-Nagel) and PrimeScript II kit (Takara), respectively. Quantitative PCR (CFX96, Bio-Rad) was performed with the following reaction parameters: 95 °C for 3 min; and 45 cycles at 95 °C for 5 s and 60 °C for 30 s with SsoAdvanced SYBR Green Supermix (Bio-Rad). Relative expression between a given sample and a reference was calculated with the E-method (Roche Applied Science; DOI:10.1038/NMETH894). The following primers were used: T1, 5′-GAAAGGGACTGGATGTTGGAG and 5′-AAATACCTTCTTCCCTGGCTG; aggrecan, 5′-TGGATCGGTCTGAATGACAGG and 5′-AGAAGTTGTCAGGCTGGTTTGG; rpl27a, 5′-TATCACCCAGGTTACTTTGGGA and 5′-ATGTCCACAGTTTATCCAGGTTG; GAPDH, 5′-CGGCAAATTCAACGGCACAGTCAA and 5′-TGGGGGCATCGGCAGAAGG; Tbp for normalization of expression analysis (Mouse Housekeeping Gene Primer Set, Takara).

### Drug infusion

Recombinant Otx2 protein produced by BL-21 bacterial cells was purified with a two-step affinity purification protocol, applied to TALON resin (Clontech) and an SP column (GE Healthcare), and the polyhistidine tag was removed by preScisson proteolytic cleavage. Flow-through fractions were quantified by immunoblotting.

Otx2 protein preincubated with polysialic acid (0.2 mg/ml), an inhibitory antibody against Otx2^11^, or vehicle solution was infused into the right V1 with an osmotic minipump (Alzet1007D, Alza) connected to a cannula that was stereotaxically implanted into mice at the ages indicated in the text. ChABC (50 U/ml, Sigma-Aldrich) or vehicle solution was injected at P24 into three different sites encompassing V1^23^. After 4–5 days of infusion, mice were processed for *in vivo* recording. DZ (2 mg/ml, Sigma-Aldrich) or vehicle solution was injected daily into both lateral ventricles, starting 1 day before MD until the day before recording^4^.

### Electrophysiology

For MD, under isoflurane anesthesia, eyelid margins were trimmed with an iris scissor and sutured. Eyes were closed for 4 days from P24–P26 or >P60, followed by *in vivo* recording under pentobarbital/chlorprothixene anesthesia. According to previous papers from several groups, no difference in recording results is present between pentobarbital^2,4,11,17,18,23,69^ and urethane anesthesia^21,24,33,70^. In particular, there is a report that reliable measurements of ocular dominance are obtained under pentobarbital rather than urethane anesthesia^71^, although both anesthetics affect inhibitory transmission^72^. Either way, unlike the specific modulation by DZ^7,58^ (see above), an acute administration of anesthetics which act as a GABA receptor agonist, globally inhibits action potentials instead of GABA transmission.

VEPs were recorded under pentobarbital/chlorprothixene anesthesia as described^17,33,69^. A tungsten electrode was inserted into the binocular zone where the maximal VEP response is located (the visual field 20° from the vertical meridian, a depth of 200–400 μm from the cortical surface). Signals were band-pass-filtered (0.1–100 Hz), amplified, and sent to a computer for analysis. At least 40 events were averaged in synchrony with the stimulus contrast reversal (ViSaGe). Transient VEPs in response to abrupt contrast reversal (100%, 1 Hz) over a range of spatial frequencies (0.05–0.7 cycles/degree) were evaluated in the time domain by measuring the peak-to-baseline amplitude of the major negative component. Visual acuity was obtained by extrapolation to 0 amplitude after correction of the noise level.

*In vivo* extracellular recordings were performed under pentobarbital/chlorprothixene anesthesia as described^2,11^ At least 20 units were recorded across the mediolateral axis (>3 spaced at 200-μm intervals, 5–7 units per penetration) to map the monocular and binocular zones. Spikes in response to a high-contrast moving bar were amplified and thresholded to count responsible signals. Peak response ratios (contralateral/ipsilateral) were assigned ocular dominance scores using a seven-point classification scheme: group 1 (purely contralateral eye response), group 2 (more than 2), group 3 (1.1 to 2), group 4 (0.9 to 1.1), group 5 (0.5 to 0.9), group 6 (less than 0.5), group 7 (purely ipsilateral eye response). The CBI was calculated from each mouse as: [(n1−n7) + 2/3 (n2−n6) + 1/3 (n3−n5) + N]/2 N, where N = total number of cells and nx = number of cells corresponding to an ocular dominance score of x. Prolonged discharge was evaluated as excessive firing beyond the receptive field as determined with a vertically moving light bar stimulus.

### Imaging analyses

Flavin imaging was performed as described^70^. Moving grating patterns (spatial frequency, 0.2 cycles/degree; speed, 5 degrees/s) were presented for 2 s in each trial at a horizontal angle of 0°. Cortical images (128 × 168 pixels) of endogenous green fluorescence (500–550 nm) in blue light (450–490 nm) were recorded from the left V1 at 9 Hz and averaged over 100 trials. Fluorescence responses were normalized as ΔF/F_0_, where F_0_ represents the average of five images immediately before stimulus onset. Amplitudes of ΔF/F_0_ were obtained with a circle window of 25 × 25 pixels.

Two-photon imaging was performed as described^70^. The excitation wavelength for fura-2 was 800 nm, and that for Venus or SR101 (Invitrogen) was 900–950 nm. Images of 256 × 256 pixels (273 × 273 µm) were recorded at 2.7 Hz and analyzed using AQUACOSMOS (Hamamatsu Photonics) and MATLAB (Mathworks). Fluorescence responses were normalized as ΔF/F_0_ (ΔF = F_0_−F), where F_0_ was obtained from averaging images for 3 s before stimulus onset. The response amplitude was the peak value during the observation window for 10 s after stimulus onset. A grating pattern (contrast, 100%; special frequency, 0.1 cycles/degree), which was pseudo-randomly moved in eight directions (from 0° to 315° in 45° steps, 25°/s velocity), was presented at a horizontal angle of 0°. The response amplitude of each neuron was calculated by averaging the responses to all eight directions.

To identify PV-cells among the recorded neurons, post-hoc immunostaining was performed. Cortical vasculature images visualized by transcardial perfusion with 10% ink-containing gelatin (Sigma) were used for reconstruction analysis. After PV immunostaining, images were obtained across the binocular zone, and distributions of Venus-labeled interneurons and vessels were matched to those in two-photon images to find the corresponding region. Finally, PV-cells were identified within the recorded plane, and responses from the PV-cells or non-PV interneurons were analyzed.

## Electronic supplementary material

Supplementary information
