# Supplementary material for: Chondroitin Sulfate Is Required for Onset and Offset of Critical Period Plasticity in Visual Cortex
Source: Sci Rep. 2017 Oct 3;7:12646. doi: 10.1038/s41598-017-04007-x (PMC5626782; doi:10.1038/s41598-017-04007-x)
Supplement: Supplementary file 1 — Supplementary information [file 41598_2017_4007_MOESM1_ESM.pdf]

Article

Chondroitin Sulfate Is Required for Onset and Offset of Critical Period Plasticity in Visual Cortex

Xubin Hou, Nozomu Yoshioka, Hiroaki Tsukano, Akiko Sakai, Shinji Miyata, Yumi Watanabe, Yuchio Yanagawa, Kenji Sakimura, Kosei Takeuchi, Hiroshi Kitagawa, Takao K Hensch, Katsuei Shibuki, Michihiro Igarashi and Sayaka Sugiyama

Figure 1J

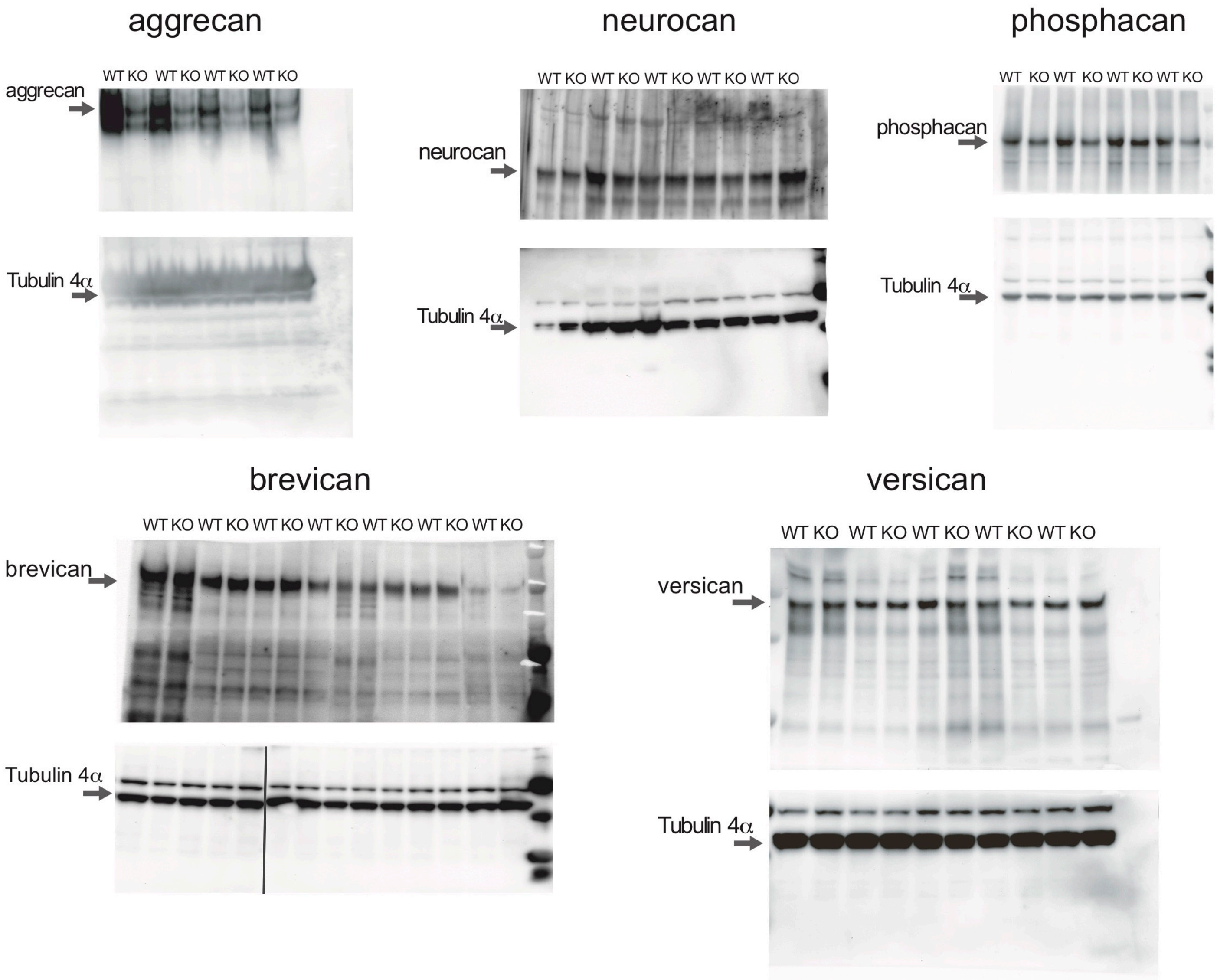

Figure 4A

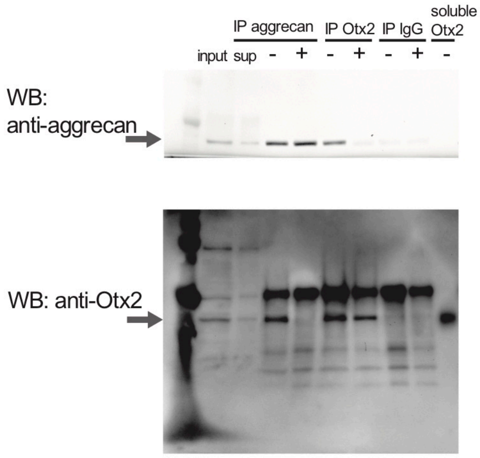

Figure 4C

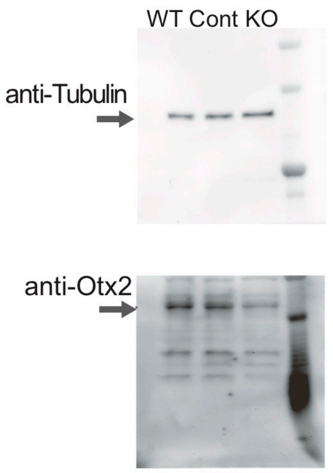

Figure 6I

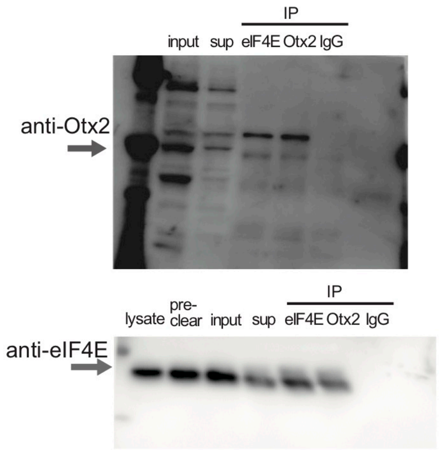

Supplementary Figure 1

Full-length images of the blots presented in the figures

In the Supplementary Figure 1J, the control (Tubulin 4α) for brevican on one blotting membrane was cut to fit the size of a heat sealable bag (*black line*) and treated together throughout blotting procedure.
